# Western diet enhances hepatic inflammation in mice exposed to cecal ligation and puncture

**DOI:** 10.1186/1472-6793-10-20

**Published:** 2010-10-19

**Authors:** Chantal A Rivera, LaTausha Gaskin, Georg Singer, Jeff Houghton, Monique Allman

**Affiliations:** 1 Department of Molecular and Cellular Physiology, Louisiana State University Health Sciences Center, Shreveport, LA USA

## Abstract

**Background:**

Obese patients display an exaggerated morbidity during sepsis. Since consumption of a western-style diet (WD) is a major factor for obesity in the United States, the purpose of the present study was to examine the influence of chronic WD consumption on hepatic inflammation in mice made septic via cecal ligation and puncture (CLP). Feeding mice diets high in fat has been shown to enhance evidence of TLR signaling and this pathway also mediates the hepatic response to invading bacteria. Therefore, we hypothesized that the combined effects of sepsis and feeding WD on TRL-4 signaling would exacerbate hepatic inflammation. Male C57BL/6 mice were fed purified control diet (CD) or WD that was enriched in butter fat (34.4% of calories) for 3 weeks prior to CLP. Intravital microscopy was used to evaluate leukocyte adhesion in the hepatic microcirculation. To demonstrate the direct effect of saturated fatty acid on hepatocytes, C3A human hepatocytes were cultured in medium containing 100 μM palmitic acid (PA). Quantitative real-time PCR was used to assess mRNA expression of tumor necrosis factor-alpha (TNF-α, monocyte chemotactic protein-1 (MCP-1), intercellular adhesion molecule-1 (ICAM-1), toll-like receptor-4 (TLR-4) and interleukin-8 (IL-8).

**Results:**

Feeding WD increased firm adhesion of leukocytes in the sinusoids and terminal hepatic venules by 8-fold six hours after CLP; the increase in platelet adhesion was similar to the response observed with leukocytes. Adhesion was accompanied by enhanced expression of TNF-α, MCP-1 and ICAM-1. Messenger RNA expression of TLR-4 was also exacerbated in the WD+CLP group. Exposure of C3A cells to PA up-regulated IL-8 and TLR-4 expression. In addition, PA stimulated the static adhesion of U937 monocytes to C3A cells, a phenomenon blocked by inclusion of an anti-TLR-4/MD2 antibody in the culture medium.

**Conclusions:**

These findings indicate a link between obesity-enhanced susceptibility to sepsis and consumption of a western-style diet.

## Background

Sepsis is a condition characterized by tachycardia, fever and leukocytosis. In severe cases, refractory hypotension and failure of multiple organ systems develops. Reportedly, the incidence of sepsis more than tripled between 1979 and 2000 [1]. It is estimated that the mortality rate due to sepsis in the general population of the United States is 28.6% [2], and the number of deaths is increasing annually [3]. Although mortality due to sepsis remains unacceptably high in the general population, rates of death among the obese patient population are even higher. In fact, death in morbidly obese subjects is approximately 7 times more likely to occur [4]. Morbidity in obese patients is also more severe. Compared to lean patients, more time is spent in intensive care units and on mechanical ventilation devices [5,6]. Causes for enhanced morbidity and mortality in obese patients remain unknown. Moreover, obesity in the United States is reaching epidemic proportions with more than 60% of adults now classified as overweight or obese. Given the difficulties and higher costs of the clinical management of these patients in addition to the growing rates of obesity, there is an urgent need to address this issue.

Various environmental factors are believed to be causally related to the obese state. Examples of these include physical inactivity and poor dietary habits, which result in caloric imbalance [7,8]. Typical eating patterns among obese populations in the US consist of foods that are high in saturated fat, sugars and cholesterol, the so-called "western diet" (WD). Thus, previous investigations have focused on elucidating the contribution of diet to the development of systemic pathologies. Cholesterol and saturated fatty acids have been linked to activation of pro-inflammatory signaling cascades in cultured macrophages [9]. These results are supported by findings *in vivo *of inflammation and endothelial dysfunction in baboons fed a high cholesterol/high saturated fat diet [10].

In an effort to understand how WD influences pathophysiology, we examined hepatic inflammation in mice fed a diet that derived a large proportion of total calories (~34%) from butter fat, which contains a significant amount of the saturated fatty acid palmitic acid (PA). To induce hepatic inflammation a murine model of cecal ligation and puncture was used to mimic sepsis. Previous studies indicate that saturated fatty acids stimulate a pro-inflammatory phenotype in cultured cells via the toll-like receptor 4 pathway [9,11,12]. Moreover, feeding mice diets high in fat has been shown to enhance evidence of TLR-4 signaling and mice deficient in TLR-4 signaling are protected from diet-induced steatohepatitis [13,14]. Since the TLR-4 pathway mediates the hepatic response to invading bacteria, we hypothesized that the combined effects of sepsis and feeding WD on TRL-4 signaling would exacerbate hepatic inflammation in septic mice.

## Results

### Weight status in mice fed western diet

**Figure 1:**
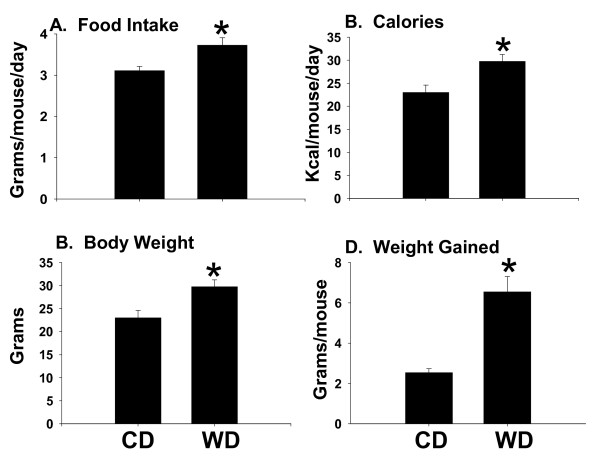
**Food intake and weight gain**. Male C57BL/6 mice were fed control (CD; n = 10) or western (WD; n = 12) diet for 3 weeks. **(A) **Food intake was monitored daily throughout the study. **(B) **Caloric intake was calculated based on the calorie content per gram of diet. For CD calories = 1345.8 Kcal/gram; WD = 3928.3 Kcal/g. **(C) **Body weight was measured at the beginning of the study just prior to feeding and at the end of the study; **(D) **average weight gained per mouse was calculated at the end of the study. Data are presented as mean SEM; *p < 0.05 versus CD-fed mice using Student's t test.

The purpose of this study was to investigate the influence of overweight status on the livers of septic mice. Accordingly, mice were fed high-calorie WD for 3 weeks prior to induction of sepsis via CLP. Average daily food intake in the WD group was 3.7 ± 0.2 grams/mouse/day and was statistically higher than amounts consumed by mice fed CD (3.1 ± 0.1 grams/mouse/day). This resulted in a significant increase in the total amount of calories consumed by mice fed WD (Figure 1 A&1B). As a result, body weight gain was more than 2-fold higher in mice fed WD (Figure 1 C&1D).

### Leukocyte trafficking is enhanced in mice fed western diet

**Figure 2:**
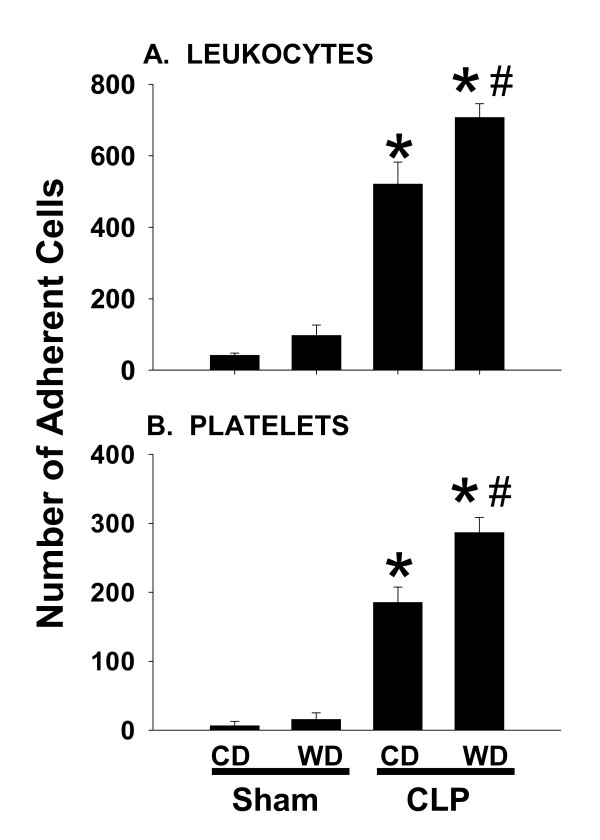
**Intravital microscopy**. Six hours after cecal ligation and puncture (CLP) mice from each dietary group were subjected to intravital microscopic analysis of leukocyte and platelet adhesion in terminal hepatic venules. A total of 3 vessels in each mouse were recorded for off-line determinations. Cells were considered adherent if they were stationary for more than 10 sec of the 1 min observation period. Data are presented as mean SEM of at least 5 mice/group. Statistical analysis was performed using two-way ANOVA and Tukey's multiple comparisons test. *p < 0.05 compared to diet-matched sham controls; #p < 0.05 compared to corresponding CD-fed mice exposed to CLP.

Intravital microscopy was used for real-time assessment of leukocyte trafficking in the liver 6 h after CLP. Leukocytes were considered to be adherent if they were stationary for more than 10 seconds of the 1 minute observation period. Feeding WD to sham controls had no significant effect on cell adhesion. In Mice fed CD, a minimal number of leukocytes were found to adhere within terminal hepatic venules in the sham operated group (Figure 2). As expected, CLP exaggerated the adherence of leukocytes (Figure 2A) and platelets (Figure 2B) in both dietary groups, a phenomenon that was significantly more pronounced in WD-fed mice. To determine if increased adherence was due to elevations in the number of circulating leukocyte in mice fed WD, whole blood samples were evaluated for leukocyte content. Results indicated that total leukocyte counts were similar between CD- (5.8 ± 1.0 × 10^6 ^cells/ml) and WD-fed mice (5.7 ± 1.7 × 10^6 ^cells/ml) prior to CLP.

### Western diet exaggerates hepatic inflammation

**Figure 3:**
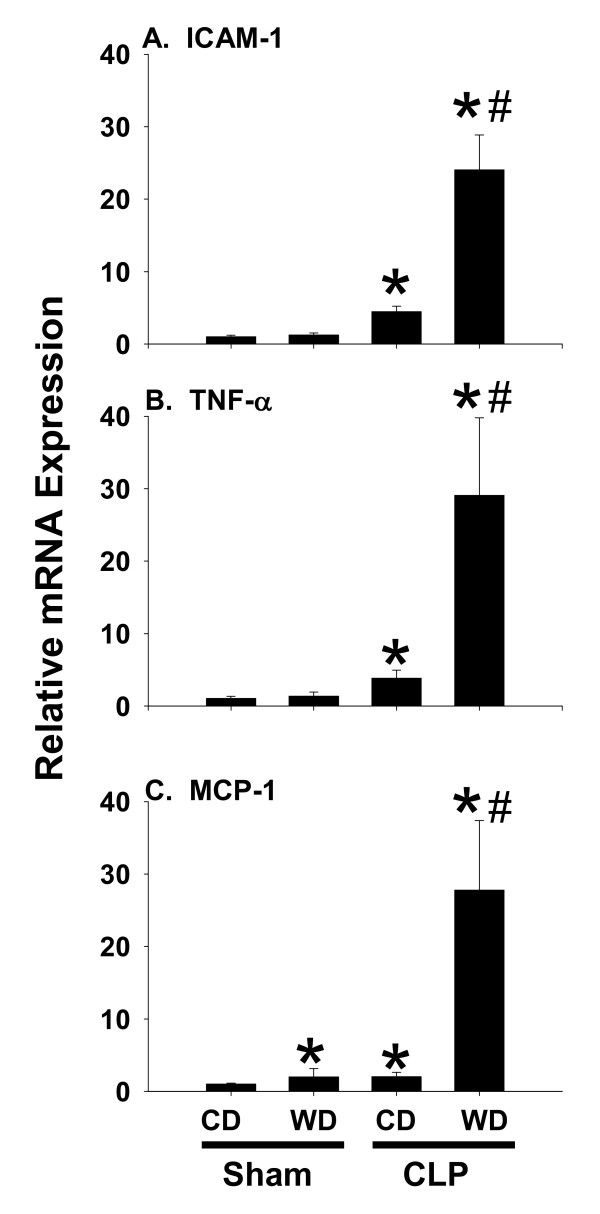
**The expression of pro-inflammatory markers**. Expression of (A) ICAM-1, (B) TNF-α and (C) MCP-1 was assessed using pre-developed assays for real-time PCR according to the manufacturer instructions (Applied Biosystems). Data are presented as mean SEM of at least 5 observations/group. Statistical analysis was performed using two-way ANOVA and Tukey's multiple comparisons test. *p < 0.05 compared to diet-matched sham controls; #p < 0.05 compared to corresponding CD-fed mice exposed to CLP.

To further index the hepatic inflammatory state total RNA was isolated to analyze expression of representative markers. Accordingly, mRNA levels of ICAM-1, TNF-α, and MCP-1 were analyzed using real-time PCR. Expression of TNF-α, and ICAM- 1 was increased by approximately four-fold 6 h after CLP in CD-fed mice (Fig 3A&3B); however, these markers were not elevated by feeding western diet in the absence of CLP. In the WD + CLP group a dramatic augmentation of TNF-α and ICAM-1 expression was observed. Expression of MCP-1 was also increased by approximately 3- and 30-fold in mice fed CD or WD, respectively following CLP. Induction of this chemokine was also enhanced significantly by feeding WD to sham control mice (Figure 3C).

### Evidence of increased toll-like receptor-4 expression

**Figure 4:**
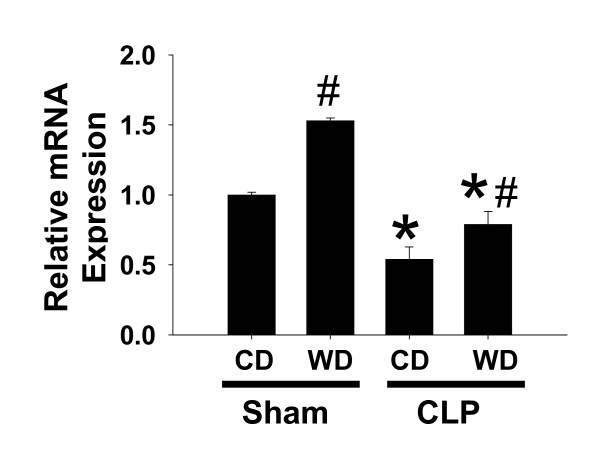
**Toll-like receptor-4 expression**. TLR-4 was assessed using pre-developed assays for real-time PCR and values were calculated using a comparative CT method according to the manufacturer instructions (Applied Biosystems). Data are presented as mean SEM of 5 observations/group. Statistical analysis was performed using two-way ANOVA and Tukey's multiple comparisons test. *p < 0.05 compared to sham controls; #p < 0.05 compared to corresponding CD-fed mice exposed to CLP.

It is well known that toll-like receptors aid in host defense against invading bacterial pathogens. In an attempt to identify possible mechanisms by which feeding WD might enhance the inflammatory response to CLP we examined the influence of WD on TLR-4 expression. As expected, a marked increase in TLR-4 mRNA was observed after feeding WD in sham mice (Figure 4). Messenger RNA levels of TLR-4 were diminished by CLP; however, expression remained significantly higher in WD-fed mice.

### Palmitic acid enhances the inflammatory response to lipopolysaccharide

**Figure 5:**
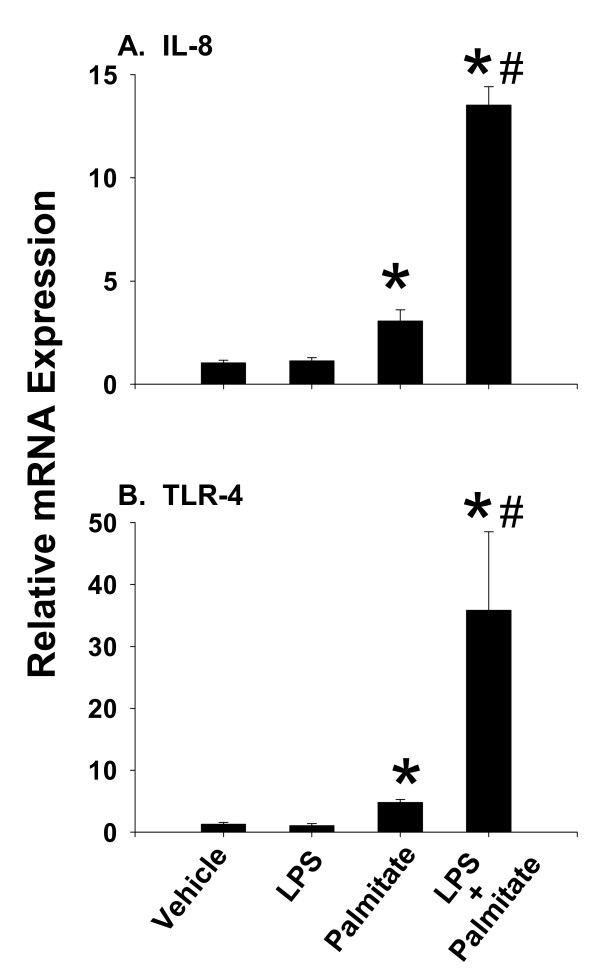
**Chemokine and TLR-4 expression in palmitic acid-treated C3A hepatocytes**. C3A hepatocytes were cultured in medium containing lipopolysaccharide (LPS) (500 pg/ml), palmitic acid (PA; 100 mM) or the combination of PA and LPS for 24h. Expression of **(A) **IL-8 and **(B) **TLR-4 was assessed using pre-developed assays for real-time PCR and values were calculated using a comparative C_T _method according to the manufacturer instructions (Applied Biosystems). Data are presented as mean SEM of 4 experiments. Statistical analysis was performed using two-way ANOVA and Tukey's multiple comparisons test. *p < 0.05 compared to vehicle control cultures; #p < 0.05 compared to PA alone.

A major component of the WD used in this study is palmitic acid (PA). Exposure of macrophages to PA has been shown to stimulate pro-inflammatory conditions and studies suggest that the response to saturated fatty acids, including PA, is mediated via the TLR-4 signaling pathway [18]. Based on findings of enhanced TLR-4 expression in mice fed WD, we hypothesized that PA augments the response to bacterial toxins (lipopolysaccharide; LPS). To address this question, the direct inflammatory response to LPS, PA or the combination of these was investigated in cultured C3A human hepatocytes. After 24 h, mRNA levels of TLR-4 and the chemokine IL-8 were measured by real-time PCR. Exposure of C3A cells to LPS or PA for 2 h did not result in notable cell death as demonstrated by the lack trypan blue uptake (data not shown). The low dose of endotoxin used in this study (500 pg/ml) did not alter expression of these parameters, whereas PA increased expression of both markers by approximately 5-fold (Figure 5). Moreover, the combination of LPS + PA further enhanced expression by 15- to 30-fold.

### Palmitic acid enhances U937 adhesion via a TLR4-dependent mechanism

**Figure 6:**
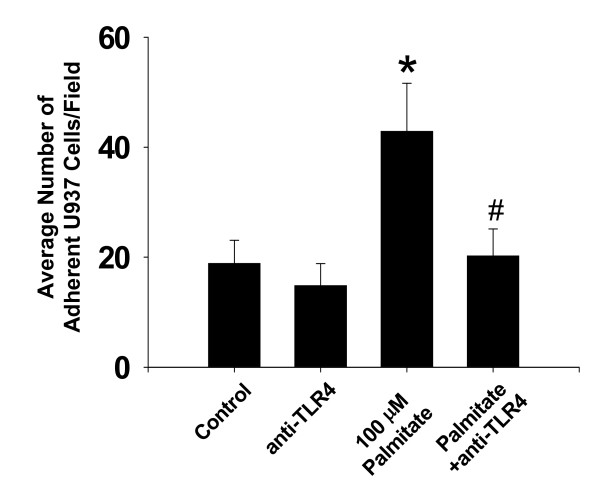
**Static adhesion of monocytes to palmitic acid -treated C3A hepatocytes**. C3A cells were treated with PA (100 mM) or PA + anti-TLR-4 blocking antibody (2 μg/ml). After 24h U937 monocytes were allowed to settle on the C3A monolayer and the number of firmly adherent cells was determined following inversion of the co-cultures. Data are presented as mean SEM of 4 experiments. *p < 0.05 compared to vehicle control cultures; #p < 0.05 compared to palmitate alone.

To investigate the potential importance of TLR-4 in the pro-inflammatory condition resulting from PA, a static adhesion assay was performed in which one experimental series included C3A cells pretreated with an anti-TLR-4/MD2 blocking antibody. Following stimulation of C3A cells with PA, U937 cells were allowed to settle on the C3A monolayer and the number of firmly adherent cells was determined following inversion of the co-cultures. A minimal number of U937 cells were observed to adhere to mock-stimulated C3A cells (Figure 6). Consistent with in vivo video microscopy results, PA potentiated adhesion by approximately 60% above control values. This response was largely blocked by addition of the TLR-4/MD2 blocking antibody.

## Discussion

Recent findings in rodent models provide insight into the influences of obesity on the pathogenesis of sepsis. For example, brain microvascular defects were found to be significantly worse in genetically obese ob/ob mice [19]. In the gut, microvascular dysfunction, inflammation and thrombocytopenia were enhanced in septic ob/ob mice and hyperphagic db/db mice when compared to their lean controls [19]. During sepsis, the liver is one of the most commonly affected organs and hepatic failure in this setting is often fatal [20]. Moreover, obese patients are likely to be more sensitive to sepsis [4-6], possibly due to the presence of hepatic steatosis or the more extensive pathology known as non-alcoholic steatohepatitis, a condition involving steatosis, inflammation, necrosis and may also include some degree of fibrosis. Thus, the present study addressed the influence of obesity specifically on the hepatic microvasculature. Approximately 72% of the daily energy intake in western cultures is provided by processed foods such as cereals, refined sugar and refined oils that would not have been available prior to the industrial revolution. Additional agricultural advances have greatly enhanced the constitutive level of saturated fatty acids in domesticated animals used for food, primarily due to grain feeding. Since genetic causes for obesity are relatively rare in humans, we chose a more clinical approach to obesity, feeding a diet with a nutrient content resembling what is consumed by humans in western cultures. Consistent with previous findings in the gut and brain of genetically obese mice, the dietary obesity model used herein exacerbated leukocyte and platelet adhesion in terminal hepatic venules. To index the inflammatory state, mRNA levels of ICAM-1 and MCP-1 were determined. Respectively, these well-characterized proteins mediate the firm adhesion of leukocytes to endothelial cells as well as hepatocytes and the chemotaxis of monocytes/macrophages. The typical expression pattern of ICAM-1, MCP-1 and the pro-inflammatory cytokine TNF-α expected in septic mice was exaggerated in mice fed WD. Since our initial evaluations of whole blood prior to CLP revealed that total leukocyte counts were similar between CD- and WD-fed mice, it is not likely that the observed enhanced adhesion was due to increased total cell numbers; however, the influence of CLP on total leukocyte counts was not evaluated.

In an attempt to uncover the molecular signal for enhanced inflammation following CLP in mice fed WD, we investigated components of TLR-4 signaling. This receptor is expressed on most cell types in the liver, including Kupffer cells, endothelial cells and hepatocytes. Our results revealed increased TLR-4 mRNA levels.. This is consistent with the idea that the inflammatory response caused by the diet is mediated through the TLR-4 signaling pathway. Indeed, several investigations have now reported a link between inflammation and TLR-4 signaling in rodents fed high fat diet and in isolated cells exposed to purified fatty acids [11,13,21,22]. Base on these findings, we hypothesized that the saturated fatty acid component of WD may be important for the development of the exaggerated inflammatory phenotype observed in septic mice. As expected, PA enhanced chemokine (IL-8) expression, TLR-4 mRNA levels and the static adhesion of monocytes to C3A hepatocytes. Static adhesion was significantly diminished in the presence of an anti-TLR-4/MD2 antibody. This supports previously published findings that saturated fatty acids activate the innate immune response via signaling through TLR-4 [9,11,22]. Recently, it was reported that the effects of saturated fatty acids could be blocked by polymyxin B, suggesting that the observed inflammatory influence of fatty acids was attributable to endotoxin [23]. These observations were generated using HEK-293 cell that were transfect with TLR-4 as this cell line does not express an endogenous form of this receptor. Thus, the exact nature of the interaction between TLR-4, fatty acids and endotoxin requires further investigation. It should be noted that CLP decreased mRNA levels of TLR-4. Diminished TLR-4 expression has also been reported in isolated cells exposed directly to endotoxin [24]; however, the reason for this down-regulation response during infection is not known.

Alternatively, fatty acids may directly modify proteins such as those involved in the inflammatory cascade. For example, it is well known that leukocytes and platelets are important mediators of the disturbances in the microvasculature that are hallmark features of sepsis [25,26]. P-selectin is an adhesion molecule that is constitutively expressed by megakaryocytes and is found at high levels on the surface of activated platelets [27]. The saturated fatty acids PA and stearic acid have both been shown to acylate P-selectin [28], which has been postulated to regulate the function and intracellular trafficking of this receptor. In addition to leukocytes we found a significant induction of platelet adhesion in terminal hepatic venules that was augmented significantly by feeding WD. Although not tested directly herein, previous observations of P-selectin acylation by PA suggest a potential mechanism for how WD might affect platelet/leukocyte adhesion and contribution to vascular defects during sepsis. The direct effects of other constituents in WD remain to be tested.

## Conclusions

In the context of the present results it is concluded that diet is causally related to the exaggerated response to sepsis observed in obese subjects. The inflammatory phenotype was partially mimicked by exposing hepatocytes to PA. The WD formulation we used also contains carbohydrates and cholesterol. Since these have also been shown to promote inflammation [10,29,30], a role in enhanced inflammation during CLP can not be ruled out.

Mortality due to sepsis in morbidly obese subjects is estimated to be 7 times more prevalent compared to mortality in lean individuals [4]. Morbidity in obese patients is also more severe [5,6]. Given the difficulties and higher costs of the clinical management of these patients in addition to the growing rates of obesity, there is an urgent need to address this issue; however, excessive body mass is often one of the criteria that excludes obese people from trials of pharmacotherapies for the treatment of sepsis. A recent phase 2 trial evaluated the efficacy of the TLR-4 antagonist eritoran tetrasodium for the treatment of sepsis in normal weight patients (~86 kg). It was determined that eritoran was beneficial in patients with the highest probability of mortality [31]. The results presented herein demonstrating an association between TLR-4 expression and exacerbated inflammation in mice fed western diet suggest that targeting the TLR signaling pathway as a therapeutic approach to the medical management of sepsis may be especially beneficial in obese patients.

## Methods

### Animal Treatment

Male C57BL/6 mice (4-6 wk) were fed *ad libitum *control diet (CD; n = 10) or western diet (WD; n = 12) that was enriched in saturated fat for 3 weeks. The components of each diet are listed in Table 1. While in the fed state, mice were subjected to cecal ligation and puncture (CLP) to induce sepsis as described previously [15]. Briefly, while under ketamine hydrochloride (150 mg/kg i.m.) and xylazine (7.5 mg/kg i.m.) anesthesia a laparatomy was performed. The cecum was exteriorized and ligated distal of the ileocecal valve then perforated three times using a 20-gauge needle (top, middle and bottom third). Cecal contents were manually extruded and spread with a sterile cotton swab. Subsequent to closure of the incision, mice received 1 ml of saline for fluid resuscitation. Mice subjected to laparatomy and exteriorization of the cecum without ligation and puncture were used as sham controls. The protocols used for handling mice were approved by the Louisiana State University Health Sciences Center Animal Care Committee and were in accordance with the guidelines set by the National Institutes of Health *Guide for Care and Use of Laboratory Animals*.

**Table 1:** Diet Compositions

	Control Diet		Western Diet	
	**g/kg**	**Kcalories/g**	**g/kg**	**Kcalories/g**
Casein	195	504	140	504
Dyetrose	311.53	0	119.28	0
Butter Fat	50	360	150	1350
Sucrose	100	400	500	2000
Cellulose	50	0	50	0
Mineral Mix	35	30.8	35	30.8
Vitamin Mix	10	39	10	39
DL-Methionine	3	12	1.12	4.48
Calcium Carbonate	4	0	2.5	0
Ethoxyquin	0.04	0	2.1	0
**Total Calories/g**		1345.8		3928.3

### Platelet isolation and infusion

The method used to isolate and fluorescently label platelets was described previously [15]. Approximately 0.9 ml of blood was collected from CD- or WD-fed donor mice via the carotid artery into 0.1 ml acid-citrate-dextrose (Sigma, St. Louis, MO). Platelet-rich plasma was obtained by centrifugation at 120 *g *for 8 min followed by spinning the plasma at 120 *g *for 3 min. The platelets were then pelleted at 735 *g *for 10 min, resuspended in 500 ml PBS (pH 7.4), and counted. The platelets were incubated for 10 min at room temperature in 90 μ M fluorochrome carboxyfluorescein diacetate succinimudyl ester (Molecular Probes, Eugene, OR), centrifuged to remove fluorochrome, and then resuspended in PBS at a concentration of 8.33 × 10^5 ^cells/ml. Labeled platelets (100 × 10^6^) were infused into the femoral vein over a period of 5 min and were allowed to circulate for 5 min before the observation period. It is estimated that labeled platelets account for approximately 10% of the total murine platelet count (assuming a 1 ml total blood volume and an average circulating platelet count of 0.8-1.2 × 10^6^/μl)

### Intravital Microscopy

To examine the effects of CLP on hepatic leukocyte trafficking, mice were re-anesthetized with xylazine and ketamine 6 hr after CLP. This early time point was chosen to minimize attrition due to mortality. Leukocytes were labeled by administering 0.02% rhodamine 6G via the tail vein. A midline incision was made to expose a small portion of liver and intravital microscopy was performed as described previously [16]. An Olympus IX71 inverted microscope equipped with a Sony DSP 3CCD color video camera was used to record each intravital experiment for offline analysis. Three non-overlapping regions were analyzed for each mouse, and each region was recorded for 2 min. Postcapillary venules between 20 and 50 μm in diameter were analyzed for rolling and adherence. Rolling cells (leukocytes and platelets) were determined visually as cells moving at a velocity notably slower by approximately 40% of the centerline velocity of the microvessel, or stationary for 2-10 seconds. Adherent cells were cells that remained stationary within a 10 second observation period.

### Total leukocyte counts

After 3 wk of feeding, a 10 μl blood sample was collected from the tail vein. Total leukocytes were counted with an electronic particle counter (Coulter Counter ZM, Coulter Corporation, Miami, Fla.) according to the manufacturer's instructions.

### Culture and Treatment

Human C3A hepatocytes (ATCC, Manassas, VA), a line sub-cloned from HepG2 cells, were grown to confluence on 48-well plates using Eagle's Minimum Essential Media (EMEM) supplemented with 10% Fetal Bovine Serum (FBS) as the culture medium. For treatment of C3A cells with palmitic acid (Sigma-Aldrich, St. Louis, MO), a 5 mM stock solution was prepared in methanol. The stock was diluted to the desired concentrations using EMEM that contained 1% BSA as a carrier. Lipopolysaccaride (LPS) from *E. Coli *(serotype 111:B4; Sigma-Aldrich, St. Louis, MO) was suspended in EMEM containing 1% BSA; the working concentration for cell treatment was 500 pg/ml. Cells were exposed to palmitate, LPS or PA + LPS for 24 h.

### Static Adhesion Assay

Confluent monolayers of C3A cells were treated with PA (100 mM) or PA + anti-TLR-4/MD2 blocking antibody (2 μg/ml) for 24 h. For static adhesion, monolayers were rinsed with PBS to remove palmitate and blocking antibody. U937 monocytes (10^5 ^cells in 100 μl PBS) were allowed to settle onto the monolayers for 500 seconds. Plates were then inverted to allow removal of non-adherent cells. After 500 seconds of inversion the number of U937 cells remaining in the viewing plane of the C3A monolayer was assessed. To distinguish between monocytes and hepatocytes, the U937 cells were labeled via incubation with Hoestch dye #3352 prior to performing the static adhesion assay. The number of adherent U937 cells was counted in 3 random fields/culture plate.

### Quantitative Real-Time Polymerase Chain Reaction

Total RNA was extracted from confluent C3A cell monolayers and 30 mg samples of liver tissue using the Qiagen RNeasy mini kit (Qiagen, Chatsworth, CA). From each sample, 500 ng of total RNA was reverse transcribed according to the manufacturer's instruction (Applied Biosystems, Foster City, CA). For real-time PCR analysis, cDNA was amplified using pre-developed assays for macrophage chemoattractant protein-1 (MCP-1), TLR-4, TNF-α, and ICAM-1 that were obtained commercially (Applied Biosystems, Foster City, CA). All reactions were performed in duplicate using an Applied Biosystems 7500 PCR System and raw data were analyzed using the Applied Biosystems Prism Sequence Detection 1.9.1 software. The amount of mRNA relative to the 18s subunit was calculated using the comparative C_t _(ΔΔC_t_) method as described previously [17].

### Statistical Analysis

Data are presented as mean ± SEM of 4-6 observations per treatment group. Statistical analysis was performed using student's t test or two-way ANOVA with Tukey's multiple comparisons test where appropriate as indicated in the figure legends; p < 0.05 was selected as the level of significance.

## Authors' contributions

CAR: Contributed the conceptual design, data acquisition, analysis and interpretation; was involved in manuscript preparation and has consented to the publication of this manuscript.

LG: Contributed toward data acquisition, analysis and interpretation; was involved in manuscript preparation and has consented to the publication of this manuscript.

GS: Contributed toward data acquisition, analysis and interpretation; was involved in manuscript preparation and has consented to the publication of this manuscript.

JH: Contributed toward data acquisition, analysis and interpretation; was involved in manuscript preparation and has consented to the publication of this manuscript.

MA: Contributed the conceptual design, data acquisition, analysis and interpretation; was involved in manuscript preparation and has consented to the publication of this manuscript.

## References

[B1] Martin GS Mannino DM Eaton S Moss M The epidemiology of sepsis in the United States from 1979 through 2000 N Engl J Med 2003 348 1546 1554 10.1056/NEJMoa022139 12700374

[B2] Angus DC Linde-Zwirble WT Lidicker J Clermont G Carcillo J Pinsky MR Epidemiology of severe sepsis in the United States: analysis of incidence, outcome, and associated costs of care Crit Care Med 2001 29 1303 1310 10.1097/00003246-200107000-00002 11445675

[B3] Vincent JL Taccone F Schmit X Classification, incidence, and outcomes of sepsis and multiple organ failure Contrib Nephrol 2007 156 64 74 full_text 17464116 10.1159/000102071

[B4] Nasraway SA Jr Albert M Donnelly AM Ruthazer R Shikora SA Saltzman E Morbid obesity is an independent determinant of death among surgical critically ill patients Crit Care Med 2006 34 964 970 10.1097/01.CCM.0000205758.18891.70 16484910

[B5] Akinnusi ME Pineda LA El Solh AA Effect of obesity on intensive care morbidity and mortality: a meta-analysis Crit Care Med 2008 36 151 158 10.1097/01.CCM.0000297885.60037.6E 18007266

[B6] Sakr Y Madl C Filipescu D Moreno R Groeneveld J Artigas A Obesity is associated with increased morbidity but not mortality in critically ill patients Intensive Care Med 2008 34 1999 2009 10.1007/s00134-008-1243-0 18670756

[B7] Bassett DR Jr Pucher J Buehler R Thompson DL Crouter SE Walking, cycling, and obesity rates in Europe, North America, and Australia J Phys Act Health 2008 5 795 814 19164816 10.1123/jpah.5.6.795

[B8] Varela-Moreiras G Controlling obesity: what should be changed? Int J Vitam Nutr Res 2006 76 262 268 10.1024/0300-9831.76.4.262 17243093

[B9] Lee JY Ye J Gao Z Youn HS Lee WH Zhao L Reciprocal modulation of Toll-like receptor-4 signaling pathways involving MyD88 and phosphatidylinositol 3-kinase/AKT by saturated and polyunsaturated fatty acids J Biol Chem 2003 278 37041 37051 10.1074/jbc.M305213200 12865424

[B10] Shi Q Vandeberg JF Jett C Rice K Leland MM Talley L Arterial endothelial dysfunction in baboons fed a high-cholesterol, high-fat diet Am J Clin Nutr 2005 82 751 759 16210703 10.1093/ajcn/82.4.751 PMC1283143

[B11] Lee JY Sohn KH Rhee SH Hwang D Saturated fatty acids, but not unsaturated fatty acids, induce the expression of cyclooxygenase-2 mediated through Toll-like receptor 4 J Biol Chem 2001 276 16683 16689 10.1074/jbc.M011695200 11278967

[B12] Maloney E Sweet IR Hockenbery DM Pham M Rizzo NO Tateya S Activation of NF-{kappa}B by Palmitate in Endothelial Cells: A Key Role for NADPH Oxidase-Derived Superoxide in Response to TLR4 Activation Arterioscler Thromb Vasc Biol 2009 29 1370 1375 10.1161/ATVBAHA.109.188813 19542021 PMC2775080

[B13] Shi H Kokoeva MV Inouye K Tzameli I Yin H Flier JS TLR4 links innate immunity and fatty acid-induced insulin resistance J Clin Invest 2006 116 3015 3025 10.1172/JCI28898 17053832 PMC1616196

[B14] Rivera CA Adegboyega P van Rooijen RN Tagalicud A Allman M Wallace M Toll-like receptor-4 signaling and Kupffer cells play pivotal roles in the pathogenesis of non-alcoholic steatohepatitis J Hepatol 2007 47 571 579 10.1016/j.jhep.2007.04.019 17644211 PMC2094119

[B15] Singer Geor Urakami Hide Specian RD Stokes KY Granger DN Platelet Recruitment in the Murine Hepatic Microvasculature During Experimental Sepsis: Role of Neutrophils Microcirc 2006 13 89 97 10.1080/10739680500466343 16459322

[B16] Singer G Houghton J Rivera CA Anthoni C Granger DN Role of LPS in the hepatic microvascular dysfunction elicited by cecal ligation and puncture in mice J Hepatol 2007 47 799 806 10.1016/j.jhep.2007.07.021 17935822 PMC2100413

[B17] Rivera CA Abrams SH Tcharmtchi MH Allman M Ziba TT Finegold MJ Feeding a corn oil/sucrose-enriched diet enhances steatohepatitis in sedentary rats Am J Physiol Gastrointest Liver Physiol 2006 290 G386 G393 10.1152/ajpgi.00229.2005 16223947

[B18] Lee JY Plakidas A Lee WH Heikkinen A Chanmugam P Bray G Differential modulation of Toll-like receptors by fatty acids: preferential inhibition by n-3 polyunsaturated fatty acids J Lipid Res 2003 44 479 486 10.1194/jlr.M200361-JLR200 12562875

[B19] Terao S Yilmaz G Stokes KY Ishikawa M Kawase T Granger DN Inflammatory and injury responses to ischemic stroke in obese mice Stroke 2008 39 943 950 10.1161/STROKEAHA.107.494542 18239178

[B20] Beal AL Cerra FB Multiple organ failure syndrome in the 1990s. Systemic inflammatory response and organ dysfunction JAMA 1994 271 226 233 10.1001/jama.271.3.226 8080494

[B21] Tsukumo DM Carvalho-Filho MA Carvalheira JB Prada PO Hirabara SM Schenka AA Loss-of-function mutation in Toll-like receptor 4 prevents diet-induced obesity and insulin resistance Diabetes 2007 56 1986 1998 10.2337/db06-1595 17519423

[B22] Suganami T Tanimoto-Koyama K Nishida J Itoh M Yuan X Mizuarai S Role of the Toll-like receptor 4/NF-kappaB pathway in saturated fatty acid-induced inflammatory changes in the interaction between adipocytes and macrophages Arterioscler Thromb Vasc Biol 2007 27 84 91 10.1161/01.ATV.0000251608.09329.9a 17082484

[B23] Erridge C Kennedy S Spickett CM Webb DJ Oxidized Phospholipid Inhibition of Toll-like Receptor (TLR) Signaling Is Restricted to TLR2 and TLR4: roles for cd14, lps-binding protein, and md2 as targets for specificity of inhibition J Biol Chem 2008 283 24748 24759 10.1074/jbc.M800352200 18559343 PMC3259833

[B24] Medvedev AE Kopydlowski KM Vogel SN Inhibition of lipopolysaccharide-induced signal transduction in endotoxin-tolerized mouse macrophages: dysregulation of cytokine, chemokine, and toll-like receptor 2 and 4 gene expression J Immunol 2000 164 5564 5574 10820230 10.4049/jimmunol.164.11.5564

[B25] Eipel C Bordel R Nickels RM Menger MD Vollmar B Impact of leukocytes and platelets in mediating hepatocyte apoptosis in a rat model of systemic endotoxemia Am J Physiol Gastrointest Liver Physiol 2004 286 G769 G776 10.1152/ajpgi.00275.2003 14715524

[B26] Russwurm S Vickers J Meier-Hellmann A Spangenberg P Bredle D Reinhart K Platelet and leukocyte activation correlate with the severity of septic organ dysfunction Shock 2002 17 263 268 10.1097/00024382-200204000-00004 11954824

[B27] Stenberg PE McEver RP Shuman MA Jacques YV Bainton DF A platelet alpha granule membrane protein (GMP-140) is expressed on the plasma membrane after activation J Cell Biol 1985 101 880 886 10.1083/jcb.101.3.880 2411738 PMC2113718

[B28] Magee AI Lipid modification of proteins and its relevance to protein targeting J Cell Sci 1990 97 Pt 4 581 584 2077033 10.1242/jcs.97.4.581

[B29] Spruss A Kanuri G Wagnerberger S Haub S Bischoff SC Bergheim I Toll-like receptor 4 is involved in the development of fructose-induced hepatic steatosis in mice Hepatol 2009 50 1094 1104 10.1002/hep.23122 19637282

[B30] Thuy S Ladurner R Volynets V Wagner S Strahl S Konigsrainer A Nonalcoholic fatty liver disease in humans is associated with increased plasma endotoxin and plasminogen activator inhibitor 1 concentrations and with fructose intake J Nutr 2008 138 1452 1455 18641190 10.1093/jn/138.8.1452

[B31] Tidswell MM Tillis WM LaRosa SPM Lynn MP Wittek AEM Kao RM Phase 2 trial of eritoran tetrasodium (E5564), a Toll-like receptor 4 antagonist, in patients with severe sepsis *. [Article] Crit Care Med 2010 38 72 83 10.1097/CCM.0b013e3181b07b78 19661804

